# recountmethylation enables flexible analysis of public blood DNA methylation array data

**DOI:** 10.1093/bioadv/vbad020

**Published:** 2023-02-20

**Authors:** Sean K Maden, Brian Walsh, Kyle Ellrott, Kasper D Hansen, Reid F Thompson, Abhinav Nellore

**Affiliations:** Computational Biology Program, Oregon Health & Science University, Portland, OR 97239, USA; Department of Biomedical Engineering, Oregon Health & Science University, Portland, OR 97239, USA; Computational Biology Program, Oregon Health & Science University, Portland, OR 97239, USA; Department of Biomedical Engineering, Oregon Health & Science University, Portland, OR 97239, USA; Computational Biology Program, Oregon Health & Science University, Portland, OR 97239, USA; Department of Biomedical Engineering, Oregon Health & Science University, Portland, OR 97239, USA; Department of Genetic Medicine, Johns Hopkins School of Medicine, Baltimore, MD 21205, USA; Department of Biostatistics, Johns Hopkins School of Public Health, Baltimore, MD 21205, USA; Department of Biomedical Engineering, Johns Hopkins University, Baltimore, MD 21218, USA; Computational Biology Program, Oregon Health & Science University, Portland, OR 97239, USA; Department of Biomedical Engineering, Oregon Health & Science University, Portland, OR 97239, USA; VA Portland Healthcare System, Portland, OR 97239, USA; Department of Medical Informatics & Clinical Epidemiology, Oregon Health & Science University, Portland, OR 97239, USA; Department of Radiation Medicine, Oregon Health & Science University, Portland, OR 97239, USA; Computational Biology Program, Oregon Health & Science University, Portland, OR 97239, USA; Department of Biomedical Engineering, Oregon Health & Science University, Portland, OR 97239, USA; Department of Surgery, Oregon Health & Science University, Portland, OR 97239, USA

## Abstract

**Summary:**

Thousands of DNA methylation (DNAm) array samples from human blood are publicly available on the Gene Expression Omnibus (GEO), but they remain underutilized for experiment planning, replication and cross-study and cross-platform analyses. To facilitate these tasks, we augmented our recountmethylation R/Bioconductor package with 12 537 uniformly processed EPIC and HM450K blood samples on GEO as well as several new features. We subsequently used our updated package in several illustrative analyses, finding (i) study ID bias adjustment increased variation explained by biological and demographic variables, (ii) most variation in autosomal DNAm was explained by genetic ancestry and CD4+ T-cell fractions and (iii) the dependence of power to detect differential methylation on sample size was similar for each of peripheral blood mononuclear cells (PBMC), whole blood and umbilical cord blood. Finally, we used PBMC and whole blood to perform independent validations, and we recovered 38–46% of differentially methylated probes between sexes from two previously published epigenome-wide association studies.

**Availability and implementation:**

Source code to reproduce the main results are available on GitHub (repo: recountmethylation_flexible-blood-analysis_manuscript; url: https://github.com/metamaden/recountmethylation_flexible-blood-analysis_manuscript). All data was publicly available and downloaded from the Gene Expression Omnibus (https://www.ncbi.nlm.nih.gov/geo/). Compilations of the analyzed public data can be accessed from the website recount.bio/data (preprocessed HM450K array data: https://recount.bio/data/remethdb_h5se-gm_epic_0-0-2_1589820348/; preprocessed EPIC array data: https://recount.bio/data/remethdb_h5se-gm_epic_0-0-2_1589820348/).

**Supplementary information:**

Supplementary data are available at *Bioinformatics Advances* online.

## 1 Introduction

DNA methylation (DNAm) is the most commonly studied epigenetic mark, and most public DNAm array samples are generated from blood (Maden *et al.*, 2021c). In prior work (Maden *et al.*, 2021c), we conducted comprehensive cross-study analyses of human DNAm array studies with raw data deposited on the Gene Expression Omnibus (GEO) (Barrett *et al.*, 2012; Edgar *et al.*, 2002), the largest archive of publicly available array data. We confined attention to the HumanMethylation450K (HM450K) platform introduced by Illumina in 2012. HM450K arrays profile 485 577 CpG loci concentrated in protein-coding genes and CpG island regions (Bibikova *et al.*, 2011; Sandoval *et al.*, 2011). We found that: (i) a subset of Illumina’s prescribed BeadArray quality metrics explained most quality variances; (ii) samples clustered by tissue and cancer status in a principal component analysis (PCA) of autosomal DNAm; and (iii) subsets of CpG probes showed high tissue-specific DNAm variation among seven normal tissues. We further released the recountmethylation Bioconductor package (Huber *et al.*, 2015) along with uniformly processed data compilations pairing DNAm with harmonized metadata labels for age, sex, tissue and disease state.

The initial recountmethylation release left open two important issues. First, the prevalence of raw data from the newer EPIC platform (Pidsley *et al.*, 2016) is rapidly increasing while our initial data compilation included only samples run on the older HM450K platform. Second, several practical research concerns were not accommodated in the initial package release, including how to leverage public array data compilations to determine the required number of samples to test a new hypothesis, how to account for confounding factors in cross-study analyses and how to leverage public data to independently validate previously published differentially methylated probes (DMPs) and identify subsets of high-confidence biomarker candidates.

We address these outstanding issues in this article using novel cross-study and cross-platform analyses, confining attention to normal human blood samples. Blood DNAm is often probed in epigenome-wide association studies (EWAS) to discover, test and validate biomarkers (Li *et al.*, 2012; Locke *et al.*, 2019; Mikeska and Craig, 2014) for diseases, such as type II diabetes (Bacos *et al.*, 2016; Dayeh *et al.*, 2016; Willmer *et al.*, 2018), obesity (Samblas *et al.*, 2019), non-alcoholic fatty liver disease (Hyun and Jung, 2020), asthma (Thibeault and Laprise, 2019) and dementia (Fransquet *et al.*, 2020), as well as colorectal (Alizadeh-Sedigh *et al.*, 2021; Dong and Ren, 2018; Jensen *et al.*, 2019; Lin *et al.*, 2021), esophageal (Yu *et al.*, 2018), breast (Guan *et al.*, 2019), pancreatic (Henriksen and Thorlacius-Ussing, 2021) and head-and-neck (Danstrup *et al.*, 2020) cancers. It is widely used to study biological aging (Haftorn *et al.*, 2021; Hannum *et al.*, 2013; Horvath, 2013; Merid *et al.*, 2020) and normal tissue epigenetics (Åsenius *et al.*, 2020; Huang *et al.*, 2016), including development and function of the immune system (Parveen and Dhawan, 2021). Recent work studied how gestational age-related differential DNAm relates to fetal health and disease risk (Mayne *et al.*, 2017; Merid *et al.*, 2020). Further, cord blood DNAm is increasingly used to precisely quantify fetal gestational age (Bohlin *et al.*, 2016; Haftorn *et al.*, 2021; Knight *et al.*, 2016; Lee *et al.*, 2019), which may lead to improvement in the efficacy of prenatal screening (Fung *et al.*, 2020). In addition, many software tools were trained and designed for use with blood DNAm data; these included methods for cell-type deconvolution (Houseman *et al.*, 2012; Koestler *et al.*, 2016; Salas *et al.*, 2022), inference of population genetic structure and shared genetic ancestry (Rahmani *et al.*, 2017) and power analyses (Graw *et al.*, 2019).

DNAm differences between sexes have been observed in mouse (Masser *et al.*, 2017) and multiple human tissues including brain (Masser *et al.*, 2017), pancreas (Hall *et al.*, 2014), nasal epithelium (Nino *et al.*, 2018), cord blood (Maschietto *et al.*, 2017) and whole blood (Grant *et al.*, 2021; Inoshita *et al.*, 2015). These DNAm differences can impact insulin secretion (Hall *et al.*, 2014), risk of disease (Maschietto *et al.*, 2017; Nino *et al.*, 2018) and biological age (Masser *et al.*, 2017). Using cross-study and cross-platform compilations of whole blood and peripheral blood mononuclear cells (PBMC), we performed novel independent validation of previously published sets of probes with differential methylation between the sexes (a.k.a. ‘sex DMPs’) from two previous studies in whole blood (Grant *et al.*, 2021; Inoshita *et al.*, 2015).

## 2 Results

### 2.1 12 537 normal blood samples spanning 3 sample types were incorporated into recountmethylation

**Fig. 1.**
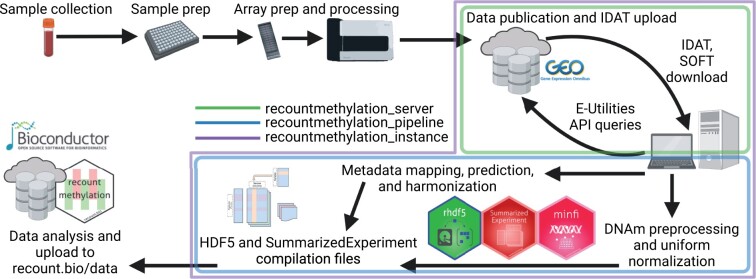
Workflow to obtain public DNAm array data from GEO. Collection, preparation and processing of array samples (top left) as well as publication of GEO datasets were performed by other investigators (top right). We downloaded raw intensity data (IDATs) and metadata (SOFTs; top right), processed GEO metadata (middle) and DNAm signals (bottom right) into HDF5-based data formats (bottom middle), and finally updated our server and the recountmethylation Bioconductor package (bottom left). Color outlines indicate data access and processing using tools we developed [green=recountmethylation_server (Maden *et al.*, 2021a), blue=recountmethylation_pipeline (Maden *et al.*, 2021b), green=recountmethylation_instance (Maden *et al.*, 2022)]. Diagrams were created with BioRender.com

**Fig. 2.**
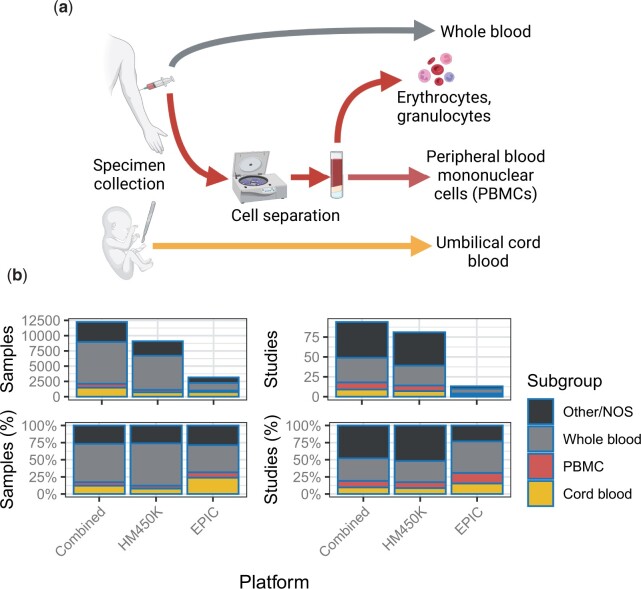
Blood specimen collection and DNAm array data availability by sample type. (**a**) Blood sample collection and handling prior to upload to GEO. (**b**) Barplot summaries of available samples (left) and studies (right), showing counts (top) and percentages (bottom) of blood sample types (black=other/not otherwise specified, gray=whole blood and red=PBMCs and yellow=cord blood). Bar heights indicate the aggregate sample group ‘all’. Diagrams were created with BioRender.com

We uniformly processed raw intensity data generated on the HM450K or EPIC platforms for 68 758 samples available on GEO before March 31, 2021 (Fig. 1, Section 4) (Aryee *et al.*, 2014; Triche *et al.*, 2013). We narrowed focus to 12 537 normal human blood samples from 63 studies, each of which had ≥10 samples after quality control. After harmonizing metadata across studies, we found these samples were predominantly of three types (Fig. 2a): whole blood, umbilical cord blood (a.k.a. ‘cord blood’) and ‘PBMC’. Each blood sample type included ≥245 samples from ≥2 studies per respective platform (Fig. 2b and Supplementary Table S1).

Whole blood was distinguishable from PBMC from erythrocyte and granulocyte DNA, as these cell types are removed during PBMC preparation (Murray and Rajeevan, 2013) (Fig. 2a). However, PBMC granulocyte proportions showed strong study-specific trends (Supplementary Fig. S1). We further observed slight-to-moderate but highly significant correlations between estimated granulocyte proportions and quality metrics (Supplementary Table S2).

We subsequently updated our Bioconductor package recountmethylation (Maden *et al.*, 2021c) to facilitate cross-study and cross-platform analyses of the blood samples. The package’s new features permit search for samples with DNAm profiles similar to a query sample (Malkov and Yashunin, 2018), inference of shared genetic ancestry (Rahmani *et al.*, 2017) and novel power analyses (Graw *et al.*, 2019). These features are explained in package vignettes. Further, a new recountmethylation_instance Snakemake workflow available on GitHub (Maden *et al.*, 2022) allows users to create their own compilations of public DNAm array data on GEO (Mölder *et al.*, 2021), with the functionality to customize output data types and attributes predicted from GEO metadata. As shown below, our resources enable identification of biomarker candidates, independent validation and replication of previous research, experiment planning and more.

Since the analyses contained in this article were performed, we have released updated recountmethylation compilations. The resource now spans all 93 306 HM450K and 38 122 EPIC samples with IDATs available on GEO before October 16, 2022.

### 2.2 Study ID adjustment increased variation explained by biological and demographic variables

We conducted simulations investigating the impact of bias correction by study ID, a surrogate for technical confounders (Maden *et al.*, 2021c). Three DNAm values were modeled in multiple regressions: (i) unadjusted DNAm, (ii) uniform adjustment on five randomly selected studies (a.k.a. ‘adjustment 1’) and (iii) exact adjustment on 2–4 randomly selected studies (a.k.a. ‘adjustment 2’). Regression models 2 and 3 were compared to test whether two distinct study ID bias adjustment strategies had comparable outcomes. We determined the fraction of explained variance (FEV) for each of 13 variables from ANOVA, yielding three results per variable per repetition of the simulation (Section 4). Total non-residual variances almost invariably decreased after applying either of the two study ID adjustment strategies (Supplementary Fig. S3a, median fractions of non-residual variances, adjusted over unadjusted, adj. 1 = 6.88e−1, adj. 2 = 6.84e−1). Variance reduction magnitudes were identical across adjustment strategies, with the exception of a few outlying models from Adjustment 1 simulations (Supplementary Fig. S3b).

**Fig. 3.**
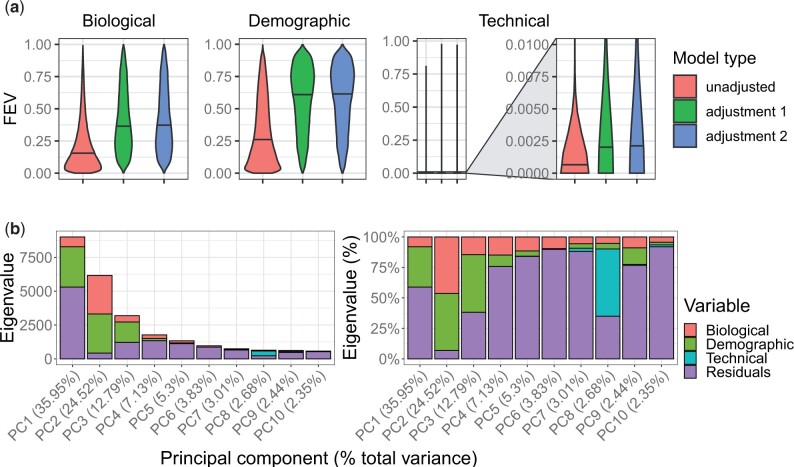
Variance analyses of study bias adjustments and principal components. (**a**) Distributions of FEV. Violin plots show results grouped by three variable categories (plot titles, one of biological on left, demographic in middle, or technical on right), and color fills show model type (pink=Adjustment 1 or adjustment on five studies, green=Adjustment 2 or adjustment on 2–4 studies and blue=unadjusted). (**b**) Autosomal DNAm PCA results across normal blood samples. Stacked barplot *y* axes show the eigenvalue magnitudes at left and percentages at right for the top 10 components on the *x* axes. Fill colors indicate magnitudes of component sum of squared variances explained by variable categories (red=biological, green=demographic, blue=technical and purple=residuals). Variables were grouped into three categories: (i) biological (blood sample type and cell type); (ii) demographic (age, sex and two genetic ancestry components); and (iii) technical (platform and study ID)

We categorized variables as biological (e.g. six predicted blood cell-type fractions), demographic (e.g. predicted sex, age and genetic ancestry), or technical (e.g. platform, where applicable). Across all three variable categories, FEV increased relative to unadjusted models after either adjustment strategy, and FEV distributions were far more similar among adjusted models than between adjusted and unadjusted models (Fig. 3a). The largest median FEV differences were observed for demographic variables, while the smallest were observed for technical variables. Among individual variables, median FEV was <0.1 across most models and variables, where study ID showed the maximum median FEV of 0.47 for unadjusted DNAm. After either adjustment, study median FEV decreased drastically to ≤2e–3, while median FEV for all remaining variables increased (Supplementary Table S3).

Because performing compilation-wide corrections on study ID substantially increased variation explained by biological and demographic variables, we included Beta-values under our adjusted models in recountmethylation for reuse in cross-study analyses.

### 2.3 Most DNAm variation was explained by predicted genetic ancestry and predicted cell composition

To better understand key sources of variation in compiled blood data, we performed PCA on normalized (Triche *et al.*, 2013), study ID-corrected autosomal DNAm, followed by ANOVA on regressions with 13 variables categorized as biological, demographic, or technical (Section 4). While most variation was residual across most components, explained variation was mainly from demographic variables at Components 1 and 3, biological variables at Components 4, 5, 6 and 10, and from technical variables at Component 8, and split between demographic and biological variables at Component 2 (Fig. 3b). Most explained variation from demographic variables was from genetic ancestry in the first component, while CD4+ T-cell fraction explained substantial biological variation across remaining top components (Supplementary Fig. S4). The top two principal components showed samples clustered largely independent from sample type and platform labels, but showed distinct gradient patterns for genetic ancestry, CD8+ T-cells, CD4+ T-cells and B-cells (Supplementary Fig. S5).

### 2.4 Dependence of statistical power on sample size was similar across blood sample types

**Fig. 4.**
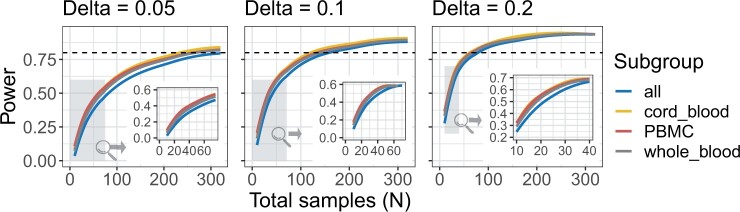
Results of power analyses with the simulation-based pwrEWAS method (Graw *et al.*, 2019). Curves indicate tradeoffs between power on the *y* axes and total samples *N* on the *x* axes, across three delta values (0.05 at left, 0.1 at middle and 0.2 at right). Curve colors show results for each sample type (line colors, blue=all, yellow=cord blood, red=PBMCs and gray=whole blood). Dotted horizontal lines show the 0.8 (80%) target power. Grayed regions and insets show magnifications of regions with low *N*

We conducted power analyses on the blood samples included in recountmethylation by applying the simulation-based pwrEWAS approach (Graw *et al.*, 2019) (Section 4). To attain ≥80% power to detect DMPs between two groups of roughly equal size, the *N* estimated total samples required were similar across sample types, where N≈300 samples at mean Beta-value difference between groups δ=0.05, N≈150 samples at δ=0.1 and N≈80 samples at δ=0.2. We assumed an FDR threshold of 5%. Outcomes were similar within each of the whole blood, cord blood and PBMC groups, but they were worse when including all blood samples, likely due to greater sample heterogeneity (Fig. 4).

Our results suggest fewer samples are necessary than the results of Graw *et al.* (2019), where adult PBMCs showed ≥80% power with N=220 samples at δ=0.1. Further, an independent power analysis using whole-blood EPIC arrays (Mansell *et al.*, 2019) found 85% of probes had >80% power with N=200 and δ=0.1, although their FDR cutoff value of 15% was less stringent than our cutoff value of 5%.

### 2.5 Sex-specific differences in estimated blood cell fractions were consistent across sample types

We investigated differences in blood cell proportions between sexes after correcting for sources of confounding (see above, Section 4). When we compared males with females using all four combined blood sample types, we found slight (<3%) but significant (*P*-adjusted ≤1.7e−7) differences in five of six cell types—or every blood cell type except monocytes. Mean proportions of CD4+ T-cells, natural killer cells and B-cells were higher in males, while mean proportions of CD8+ T-cells and granulocytes were higher in females. Comparisons by blood sample type were similar in terms of the directionality and magnitude of mean cell proportion differences by sex. PBMC and whole blood showed the most significant differences (*T*-test *P*-adjusted, Benjamini–Hochberg method, <1e−3), where females had greater mean proportions of granulocytes and monocytes (≥3.1%), and males had greater mean proportions of (≥1.7%) CD8+ T-cells, CD4+ T-cells and B-cells (Supplementary Fig. S6 and Supplementary Table S4).

### 2.6 38% of sex DMPs from a previously published EWAS study were replicated in either whole blood or PBMC

**Fig. 5.**
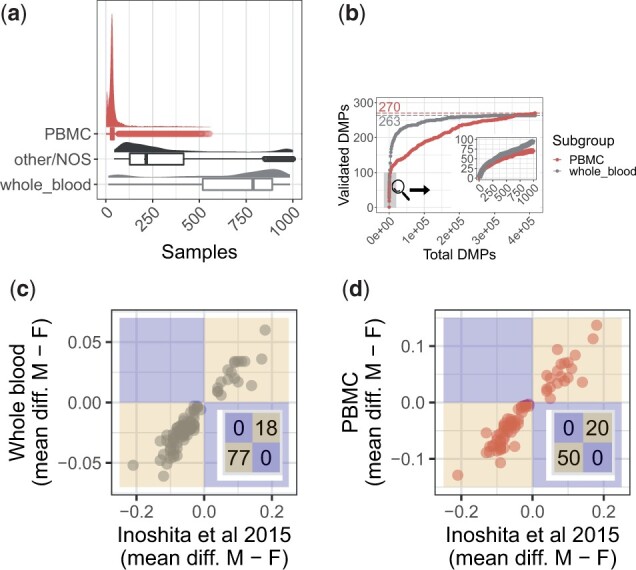
Replication of sex DMPs from Inoshita *et al.* (2015) (a.k.a ‘Inoshita et al 2015’) using cross-study compilations of whole blood and PBMC. (**a**) Sample label distributions among the 1000 nearest neighbors from querying Inoshita *et al.* (2015) samples, where density and box plots show returned frequencies of three sample labels (red=PBMCs, black=other/not otherwise specified and gray=whole blood). (**b**) Concordance of Inoshita *et al.* (2015) DMPs on the *y*-axis among the top significant compilation DMPs on the *x*-axis, ranked on *P*-values, for whole blood in gray and PBMCs in red. The zoom shows the top 1000 DMPs, and colored dotted lines and colored numbers indicate total DMPs from Inoshita *et al.* (2015) (Section 4). (**c and d**) Mean beta-value differences (male–female) at Inoshita *et al.* (2015) DMPs on *y* axes and in (c) whole blood and (d) PBMC compilations. Region colors show direction agreement in gold and disagreement in blue, and insets show DMP counts by region

We queried a search index of blood autosomal CpG DNAm, which is included in the updated recountmethylation resource, for each of the 113 whole-blood samples from Inoshita *et al.* (2015). In the process, we quantified the similarity of queried sample methylation profiles to other samples by analyzing the *k* nearest neighbors returned (Section 4). Among the 1000 nearest neighbors returned per queried sample, the whole-blood label was common while the PBMC label was rare (Fig. 5a), in agreement with Methods in Inoshita *et al.* (2015) describing the queried samples as ‘peripheral whole blood’. This greater similarity to compiled whole blood may reflect greater similarity in subject ages, cell composition (Murray and Rajeevan, 2013) and/or genetic ancestry (Supplementary Fig. S7), and we corrected for these potential confounders in regressions for identifying sex DMPs from either compilation (Section 4).

We next considered the threshold of the top 1000 most significant DMPs from whole blood and PBMC. We set this threshold because these DMPs captured the long tail of high between-sex DNAm differences for each tissue (Supplementary Fig. S8a–d), and because of less replication divergence between tissues compared to less stringent thresholds observed from our concordance at the top analysis (Fig. 5b). (112/292 =) 38% of sex DMPs from Inoshita *et al.* (2015) were replicated in either whole blood or PBMC (Fig. 5b). Of these, 42 were only replicated in whole blood, 17 were only replicated in PBMC and 53 were replicated in both tissues (Supplementary Fig. S9a). Further, (250/544 =) 46% of whole-blood sex DMPs independently reported in Grant *et al.* (2021) overlapped DMPs in whole blood or PBMC. However, just 26 sex DMPs appeared in all of PBMC, whole blood, Inoshita *et al.* (2015) and Grant *et al.* (2021). Mean (normalized) Beta-value was typically higher in females than males in both whole blood (81−93% of DMPs) and PBMC (68% of DMPs). There was 100% agreement in mean Beta-value differences between males and females among the subset of replicated DMPs in each compilation (Fig. 5c and d). Cytosine- and guanine-rich regions are known as CpG islands (Bird, 2002; Deaton and Bird, 2011; Gardiner-Garden and Frommer, 1987; Illumina, 2016; Takai and Jones, 2002), and a substantial number of replicated sex DMPs mapped at or proximal to a CpG island (84/112 = 75%). The most significant of these DMPs (*P*-adjusted <5.1e−47, Bonferroni method) mapped to a variety of functional gene regions, including two body-mapping DMPs (cg26355737 and cg04946709) at *TFDP1* and *LOC644649*, and one promoter-mapping DMP (cg09066361) at *GRM8*.

## 3 Discussion

We analyzed DNAm array data from the three most prevalent blood sample types in the GEO database and updated the recountmethylation Bioconductor package to make reproducible (Beaulieu-Jones and Greene, 2017; Heil *et al.*, 2021) cross-study and cross-platform analyses of these data easier. Since HM450K and EPIC data continue to accumulate rapidly on GEO, we further developed the recountmethylation_instance Snakemake workflow to enable semi-automated compilation of the DNAm array data on GEO (Maden *et al.*, 2022).

We replicated 38% of sex DMPs from Inoshita *et al.* (2015) and 46% of sex DMPs from Grant *et al.* (2021) using independent whole blood and PBMC compilations. These rates were similar to prior studies of sex DNAm differences, including a 38% validation rate of cord blood sex DMPs between two independent cohorts (Maschietto *et al.*, 2017), and 44% validation rate of genes in nasal epithelium with DNAm differences by sex (Nino *et al.*, 2018). These results could represent a baseline expectation for replication or independent validation rate of DMPs for sex, and potentially other variables, across independent EWAS.

Our work has several limitations. First, we excluded blood spots from our analyses due to insufficient raw DNAm array data available from GEO, although this blood sample type accounts for a substantial fraction of publicly available data from younger subjects. Another limitation related to data availability is that far fewer blood samples were available for the EPIC platform compared to HM450K as of March 31, 2021. The larger EPIC platform could help expand analyses to new genome regions and clarify regional DNAm signals at CpG islands and genes. The pwrEWAS method assumes a technical detection threshold of Beta-value = 0.01 by default, and using this threshold ensures our findings are relevant for both single-study and cross-study analyses. However, this technical threshold likely should be lowered if the study being planned involves cross-study analyses using study ID bias correction, because we found this correction reduced explained variances (section Study ID adjustment increased variation explained by biological and demographic variables) and resulted in lower between-group differences in our sex DMP cross-study analysis compared to the single-study discovery EWAS (Section 38% of sex DMPs from a previously published EWAS study were replicated in either whole blood or PBMC). A further shortcoming of our analyses is that we did not investigate the influence of SNPs on DNAm, both proximally and distally. We note, however, that recountmethylation includes a feature to filter out probes overlapping known SNPs, as described in a package vignette. We also did not quantify nucleated red blood cells, a cell type with a highly distinctive DNAm profile characteristic of cord blood samples (Gervin *et al.*, 2019). We intend to perform this quantification in a future version of recountmethylation. Finally, we did not conduct orthogonal or wet-lab validation of replicated sex DMPs. Such steps would be essential to narrow biomarker candidates and elucidate biological mechanisms explaining differential DNAm.

Our work invites further exploration in several directions. First, future studies could apply our cross-study and cross-platform compilation of cord blood samples to validate other independent EWAS, such as Solomon *et al.* (2022), a recent meta-analysis of cord blood autosomal DNAm. Second, our linear correction for study-specific bias could be compared to alternative approaches, such as embedding alignment methods, which have been used to harmonize transcriptomics data across disparate platforms and data sources (Butler *et al.*, 2018). Third, the population diversity encompassed by our cross-study compilations may permit more nuanced array-based studies of relationships between genetic variants and DNAm than previously possible. Fourth, our cross-study and cross-platform approach could be applied to other tissues with high prevalence among public datasets, such as brain (Maden *et al.*, 2021c). Finally, our DNAm array data compilations could be expanded to include public bisulfite sequencing samples from the Sequence Read Archive (Leinonen *et al.*, 2011), and these new samples can help clarify the genome region specificity and phased DNAm patterns proximal to significant DMPs and biomarker candidates (Noble *et al.*, 2021; Pidsley *et al.*, 2016; Wang *et al.*, 2015). A future update of recountmethylation may include such samples.

## 4 Methods

### 4.1 Compiling recent public DNAm array data across platforms

DNAm array data were identified, downloaded and processed using the recountmethylation_instance v0.0.1 (Maden *et al.*, 2022) Snakemake workflow. It comprises the recountmethylation_server v1.0.0 (Maden *et al.*, 2021a) and recountmethylation.pipeline v1.0.0 (Maden *et al.*, 2021b) tools, which we used previously to compile HM450K data (Maden *et al.*, 2021c). We uniformly processed samples into cross-study and cross-platform data compilations. Data were from samples run on the HM450K (Bibikova *et al.*, 2011) and EPIC (Pidsley *et al.*, 2016) DNAm array platforms and available on GEO by March 31, 2021. Sample DNAm was normalized using out of band signal (a.k.a. ‘noob-normalization’) (Triche *et al.*, 2013). Compilations paired normalized DNAm fractions, or Beta-values, with harmonized sample metadata for 68 758 cumulative samples for which raw image data files, or IDATs, were available as gzip-compressed Supplementary files.

Compilations were stored as Hierarchical Data Format 5 (HDF5)-based SummarizedExperiment files generated using the HDF5Array v1.18.0 and rhdf5 v2.34.0 R/Bioconductor packages (Fischer *et al.*, 2020; Pagès, 2021). These formats used DelayedArray v0.18.0 (Pagès *et al.*, 2021) to support rapid access, summaries and filters. For most analyses, DNAm data were merged across platforms for the 453 093 CpG probes (Pidsley *et al.*, 2016) they shared. We made compiled data available online at https://recount.bio/data/gr-gseadj_h5se_hm450k-epic-merge_0-0-3/.

### 4.2 Prediction and harmonization of sample metadata

We generated harmonized sample metadata from heterogeneous metadata mined from SOFT files accompanying GEO studies. We wrote regex terms to detect keywords in metadata-containing files, and we mapped detected terms to controlled vocabularies under ‘tissue’, ‘disease’ and other categories, as described in Maden *et al.* (2021c) and Lowe and Rakyan (2013). The suitability of regex patterns for capturing informative metadata terms was spot-checked across studies and iteratively updated to more precisely map terms and avoid erroneous mappings. We further predicted sample types from mined metadata using the method from Bernstein *et al.* (2017). To add sex annotations, we applied the getSex() function from minfi v1.37.1 R package with argument defaults. To add six blood type cell fractions, we applied the estimateCellCounts() function from minfi with argument defaults (Aryee *et al.*, 2014; Fortin *et al.*, 2017). This applies the deconvolution method from Houseman *et al.* (2012) on the blood reference dataset originally published in Reinius *et al.* (2012) and distributed in the FlowSorted.Blood.450k v1.34.0 R package. We added age annotations using the pan-tissue epigenetic clock model from Horvath (2013), which was implemented in the wateRmelon v2.2.0 package. Finally, we calculated the top components of genetic ancestry using the EPISTRUCTURE method (Rahmani *et al.*, 2017).

We noted limited overlap among CpG probes used in the above models to predict or impute missing sample metadata fields. Genetic ancestry was predicted from 4913 probes previously found to have strong association with genetic ancestry-defining SNPs after correction for bias from factors including predicted cell-type heterogeneity (Rahmani *et al.*, 2017). Ages were obtained from 353 CpG probes previously found to robustly predict age across tissues and platforms (Horvath, 2013), of which two (cg03760483 and cg04431054) overlapped probes for genetic ancestry predictions. Blood cell-type fractions were predicted from deconvolution of cell type-specific array-wide DNAm signals. The original method on which this approach is based was previously validated using probes from the older HM27K platform (Houseman *et al.*, 2012), none of which overlapped probes used to predict either genetic ancestry or age.

### 4.3 Sample QC filters

We used metadata filters to find the three most prevalent blood sample types (whole blood, cord blood and PBMCs), and to define the aggregate type ‘all’, which includes the above types and blood samples whose specific type could not be determined from their metadata. We then performed QC with reference to prior findings from Maden *et al.* (2021c). We removed samples for which either: (i) log2 median M and U signals were both <10; or (ii) the sample failed ≥2/5 most informative BeadArray quality metrics. These metrics, described by Illumina in Illumina (2010, 2015), pertain to CpG probe chemistry and performance [also see Maden *et al.* (2021c) for details]. These filtering criteria removed 245 samples, and all but one was run on the HM450K platform. We additionally filtered PBMCs with high estimated granulocyte proportions (≥0.25), and this threshold was set to remove the long tail (93rd quantile) of the granulocyte proportion distribution across studies (Supplementary Fig. S1 and Supplementary Table S2). This removed 47 PBMC samples and left 580 remaining.

### 4.4 Simulation of study bias adjustments

We used simulations to show the impact of study ID adjustment on explained variance. As detailed in Supplementary Figure S2, simulations consisted of four steps: (i) calculate sample DNAm *M*-values from 500 CpG probes and 5 studies, selected randomly; (ii) adjust study ID across all five selected studies (i.e. ‘adjustment 1’) or subsets of 2–4 studies (i.e. ‘adjustment 2’); (iii) perform ANOVA for three models; (iv) get FEV for each variable across three models. In total, simulations used 29 028 unique CpG probes and 62 unique studies.

Multiple regression models accounted for sample type, platform, study ID, DNAm-based predictions for age, sex and six cell-type fractions and two genetic ancestry components, which were determined as described above. Variables were grouped into three categories: (i) biological (blood sample type and cell type); (ii) demographic (age, sex and two genetic ancestry components); and (iii) technical (platform and study ID). Study bias adjustments were performed using the removeBatchEffect() function from the limma v3.46.0 (Ritchie *et al.*, 2015) R package. Parallel sessions were deployed using the parallel v4.1.1 R package.

### 4.5 PCA of autosomal DNAm

We performed autosomal DNAm PCA on compiled blood samples using a reduced 1000D representation of the normalized and bias-corrected Beta-values (Kane and Nelson, 2014; Williams, 2005) obtained via feature hashing [see Maden *et al.* (2021c) for details on this approach]. For the top 10 components, we calculated FEV from ANOVA using multiple regression models containing the 13 variables from the 3 categories described above.

### 4.6 Blood autosomal DNAm search index construction

We used the hnswlib v0.5.2 Python library to make a DNAm-based search index from which one can rapidly identify the nearest samples which neighbor one or more queried DNAm profiles (Malkov and Yashunin 2018). We used the Hierarchical and Navigable Small Worlds graph algorithm implemented in hnswlib, as this was among the top performing algorithms from a recent comprehensive benchmark of search algorithms (Aumüller *et al.*, 2018). With the mmh3 v3.0.0 and numpy v1.20.1 (Harris *et al.*, 2020) Python libraries, we applied feature hashing to generate a reduced 1000D representation of each sample (Kane and Nelson, 2014; Weinberger *et al.*, 2010) of each blood sample’s noob-normalized Beta-values. The search index files are available online at https://recount.bio/data/sindex-hnsw_bval-gseadj-fh10k_all-blood-2-platforms.pickle and https://recount.bio/data/sidict-hnsw__bval-gseadj-fh10k__all-blood-2-platforms.pickle.

### 4.7 Power analyses using pwrEWAS

We used the method provided in the pwrEWAS v1.4.0 R/Bioconductor library to perform power analyses across DNAm array platforms (Graw *et al.*, 2019). Parameters for these analyses included 100 total simulations varying the total samples *N* from 50 to 850. We targeted 500 DMPs and assessed test group Beta-value differences δ of 0.05, 0.1 and 0.2.

### 4.8 Replication of whole-blood sex DMPs

We replicated sex DMPs from Inoshita *et al.* (2015), a study of whole blood from Japanese individuals, using independent compilations of whole blood and PBMC samples in recountmethylation. After filtering out sex chromosome and cross-reactive probes (Chen *et al.*, 2013), there were 375 244 CpG probes in whole blood and 375 244 CpG probes in PBMCs. After filtering for sample quality, we used data from 5980 whole-blood samples (3942 females and 2924 males) and 580 PBMC samples (357 females and 223 males). Ages tended toward young adult and middle-aged for whole blood (age, mean±SD, 39±21 years) and samples from Inoshita *et al.* (2015) (46±12 years), but were more frequently from adolescents and young adults among PBMC (25±19 years). We preprocessed DNAm *M*-values using surrogate variables analysis with the sva v3.4.0 R package (Leek *et al.*, 2021). We determined sex DMPs using coefficient *P*-values for the sex variable in multiple regressions, where regression models corrected for bias from biological (six predicted blood cell-type fractions), demographic (predicted age) and technical variables (platform and study ID).

### 4.9 Statistical analyses and visualizations

Data processing and analyses were performed using the R v4.1.0 and Python v3.7.1 programming languages (Python Core Team, 2019; R Core Team, 2021). Statistical summaries and tests were performed using base R libraries. DNAm array processing, normalization, analysis and prediction of sex and six blood cell-type fractions were performed using the minfi, minfiData and minfiDataEPIC R packages. Workflow diagrams were created using BioRender.com. Visualizations in Section 2 made use of the ggplot2 v3.3.2, grid v4.1.3, gridExtra v2.3, UpSetR v1.4.0, ggpubr v0.4.0, ggforce v0.3.3 and png v0.1-7 R packages (Gehlenborg, 2019; Wickham, 2016). *P*-value adjustments used either the Bonferroni method or the Benjamini–Hochberg method (Benjamini and Hochberg, 1995). Enrichment tests used the binom.test() base R function with the background of 453 093 total probes overlapping both array platforms (R Core Team, 2021). Supplementary scripts and functions recreating our results are available online at https://www.github.com/metamaden/recountmethylation_flexible-blood-analysis_manuscript.

### 4.10 Supplementary data, files and code

The following resources have been provided to reproduce results, figures and tables in this article:

1. The updated recountmethylation Bioconductor package is now available (https://doi.org/doi:10.18129/B9.bioc.recountmethylation). It features new functions supporting analysis of large data compilations, and new vignettes showing how to perform novel power analysis, infer genetic ancestry and more using DNAm array data.
2. Supplementary code and scripts for this article, including support for creating and querying a search index of DNAm array samples, are available in the manuscript GitHub repository (https://github.com/metamaden/recountmethylation_flexible-blood-analysis_manuscript).
3. The recountmethylation_instance Snakemake workflow is available on GitHub (Maden *et al.*, 2022). This will be useful for researchers hoping to make and update new compilations of public DNAm array data from GEO.

## Supplementary Material

vbad020_Supplementary_Data

## Data Availability

Data used in this study were publicly available and downloaded from the Gene Expression Omnibus (GEO) repository at National Center for Biotechnology Information (NCBI) website (https://www.ncbi.nlm.nih.gov/geo). Compilations of the analyzed public data can be accessed from the website recount.bio/data (preprocessed HM450K array data: https://recount.bio/data/remethdb_h5se-gm_epic_0-0-2_1589820348/; preprocessed EPIC array data: https://recount.bio/data/remethdb_h5se-gm_epic_0-0-2_1589820348/).
