# Supplementary material for: recountmethylation enables flexible analysis of public blood DNA methylation array data
Source: Bioinform Adv. 2023 Feb 20;3(1):vbad020. doi: 10.1093/bioadv/vbad020 (PMC9976962; doi:10.1093/bioadv/vbad020)
Supplement: vbad020_Supplementary_Data [file vbad020_supplementary_data.zip › supplemental_tables.pdf]

Supplementary tables

	Type	all	whole blood	cord blood	PBMC
All platforms	Studies	62	31	9	9
	Samples	12,242	6,866	1,475	627
HM450K	Studies	51	25	7	7
	Samples	9,083	5,613	722	382
EPIC	Studies	12	6	2	2
	Samples	3,159	1,253	753	245
Sex	Male (fraction)	0.44	0.43	0.52	0.37
Age (years old)	Median	30	36	0	18
	SD	25	21	0	19
	1<	2,155	21	1,475	0
	1-10	859	289	0	234
	10-20	1,640	1,352	0	87
	20-40	2,923	2,252	0	166
	40-60	2,452	1,386	0	102
	60-80	2,053	1,442	0	35
	>80	160	124	0	3

Table S1: Sample and study availability, and demographic variables (rows), by blood sample type (columns).

qc.metric	corr.spear.rho	corr.spear.punadj	p.adj.bh
nonpolymorphic.red	-0.255	9.61E-11	1.83E-09
biotin.stain.grn	-0.23	5.76E-09	3.65E-08
specificityII	0.23	5.40E-09	3.65E-08
meth.l2med	-0.216	4.85E-08	2.30E-07
specificityI.red	-0.214	6.50E-08	2.47E-07
restoration.grn	-0.17	1.94E-05	5.58E-05
target.removal.1	0.169	2.05E-05	5.58E-05
bisulfite.conv.I.grn	-0.164	3.97E-05	9.43E-05
target.removal.2	0.162	4.50E-05	9.49E-05
unmeth.l2med	-0.153	1.18E-04	2.24E-04
nonpolymorphic.grn	-0.151	1.53E-04	2.64E-04
bisulfite.conv.II	0.122	2.25E-03	3.57E-03
extension.grn	0.114	4.12E-03	6.03E-03
extension.red	0.113	4.61E-03	6.25E-03
specificityI.grn	-0.095	1.74E-02	2.20E-02
hyb.hi.med	-0.078	5.19E-02	6.16E-02
biotin.stain.red	-0.066	1.00E-01	1.12E-01
bisulfite.conv.I.red	0.029	4.73E-01	4.99E-01
hyb.med.low	-0.006	8.79E-01	8.79E-01

Table S2: Spearman correlation results comparing estimated Granulocyte proportions in PBMC samples to 19 QC metrics, including 17 BeadArray metrics prescribed by Illumina, log_2-scaled methylated signal, and log_2-scaled unmethylated signal.

	Model	Unadjusted	Adjustment 1	Adjustment 2
Technical	Study ID	0.465	0.002	0
	Platform	0.037	0.086	0.089
Biological	CD8+ T-cells	0.014	0.036	0.037
	CD4+ T-cells	0.01	0.026	0.026
	B-cells	0.013	0.035	0.035
	Gran.	0.02	0.05	0.051
	Mono.	0.005	0.013	0.013
	Natural Killer	0.009	0.024	0.024
Demographic	Sex	0.003	0.008	0.008
	Age	0.012	0.03	0.031
	G.A. PC1	0.077	0.212	0.214
	G.A. PC2	0.054	0.144	0.144

Table S3: Fraction of explained variance (FEV) medians by model type (columns) and variables (rows), across study ID bias correction simulations (Methods).

blood.subgroup	cell.type	mean.male	var.male	mean.female	var.female	ttest.stat	ttest.punadj	ttest.padj.bh
other/NOS	CD8T	0.104	0.015	0.122	0.013	-4.31	1.66E-05	9.93E-05
	CD4T	0.183	0.022	0.171	0.017	2.51	1.22E-02	1.83E-02
	NK	0.054	0.004	0.055	0.003	-0.29	7.70E-01	7.70E-01
	Bcell	0.086	0.003	0.079	0.002	4.01	6.30E-05	1.89E-04
	Mono	0.091	0.002	0.086	0.002	2.60	9.44E-03	1.83E-02
	Gran	0.502	0.033	0.51	0.03	-1.25	2.13E-01	2.55E-01
whole_blood	CD8T	0.09	0.003	0.093	0.002	-2.84	4.47E-03	5.37E-03
	CD4T	0.151	0.004	0.133	0.005	10.90	1.96E-27	2.94E-27
	NK	0.071	0.003	0.056	0.002	12.39	8.38E-35	1.68E-34
	Bcell	0.076	0.002	0.061	0.002	14.70	4.70E-48	2.82E-47
	Mono	0.083	0.001	0.083	0.001	-0.81	4.19E-01	4.19E-01
	Gran	0.554	0.013	0.594	0.015	-13.86	4.57E-43	1.37E-42
cord_blood	CD8T	0.096	0.003	0.103	0.003	-2.64	8.40E-03	2.14E-02
	CD4T	0.165	0.009	0.148	0.009	3.48	5.20E-04	3.12E-03
	NK	0.044	0.002	0.05	0.003	-2.56	1.07E-02	2.14E-02
	Bcell	0.14	0.002	0.136	0.003	1.56	1.18E-01	1.77E-01
	Mono	0.122	0.002	0.121	0.002	0.18	8.59E-01	8.59E-01
	Gran	0.469	0.028	0.476	0.03	-0.79	4.29E-01	5.15E-01
PBMC	CD8T	0.223	0.009	0.198	0.007	3.41	7.21E-04	1.08E-03
	CD4T	0.344	0.018	0.345	0.012	-0.09	9.28E-01	9.28E-01
	NK	0.121	0.015	0.093	0.007	3.09	2.16E-03	2.59E-03
	Bcell	0.158	0.004	0.139	0.003	3.71	2.31E-04	4.62E-04
	Mono	0.118	0.008	0.15	0.01	-4.03	6.48E-05	1.94E-04
	Gran	0.05	0.005	0.093	0.021	-4.95	9.54E-07	5.72E-06
all	CD8T	0.1	0.007	0.108	0.006	-13.93	4.49E-44	5.39E-44
	CD4T	0.17	0.012	0.157	0.012	17.60	3.48E-69	5.22E-69
	NK	0.065	0.004	0.057	0.003	19.32	5.86E-83	1.17E-82
	Bcell	0.091	0.003	0.078	0.003	36.69	<5.0E-100	<5.0E-100
	Mono	0.092	0.002	0.092	0.002	0.56	5.77E-01	5.77E-01
	Gran	0.506	0.03	0.53	0.034	-19.43	6.62E-84	1.99E-83

Table S4: Comparison of predicted blood cell proportions between males and females.
