# Supplementary material for: recountmethylation enables flexible analysis of public blood DNA methylation array data
Source: Bioinform Adv. 2023 Feb 20;3(1):vbad020. doi: 10.1093/bioadv/vbad020 (PMC9976962; doi:10.1093/bioadv/vbad020)
Supplement: vbad020_Supplementary_Data [file vbad020_supplementary_data.zip › supplemental_figures.pdf]

Supplementary figures

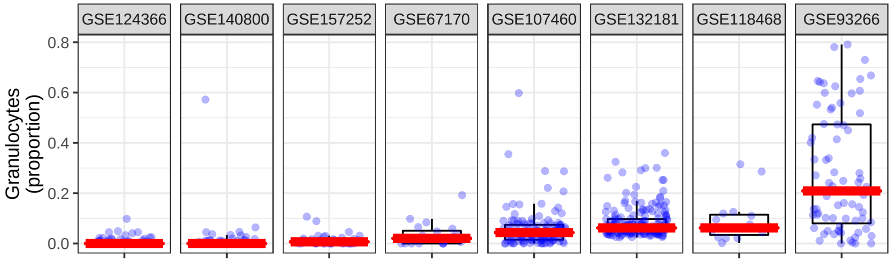

(a)

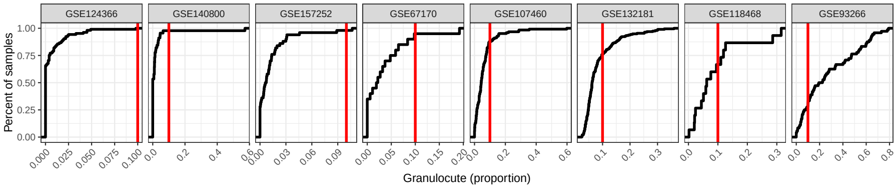

(b)

Fig. S1: Estimated Granulocytes by study in PBMC samples. (a) Aggregate distribution plots of Granulocyte proportions (y-axes) by study (x-axes, plot labels), showing a boxplot (black lines), jittered scatter plot (blue points), and median indicators (horizontal heavy red lines). (b) Cumulative empirical distribution step plots showing percent of samples (y-axes) by Granulocyte proportions (x-axes), organized by study (plot labels). Vertical red lines indicate where Granulocyte proportion equals 0.1 or 10%.

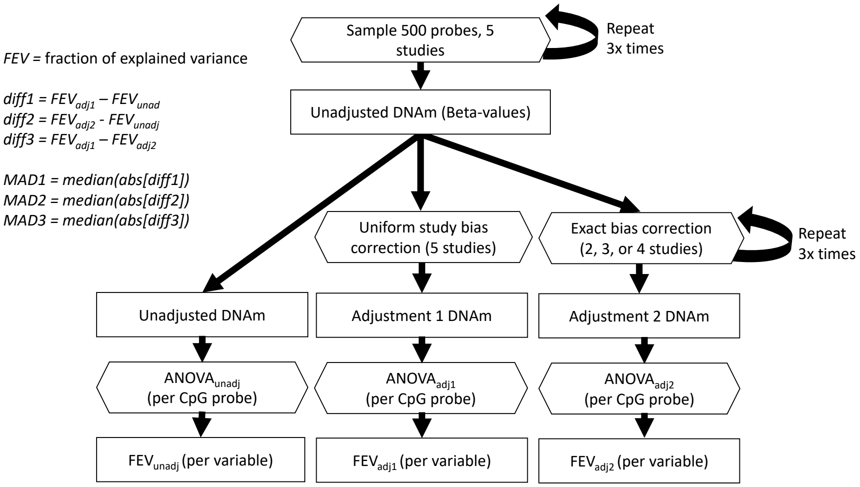

Fig. S2: Workflow diagram to simulate the impact of GSE bias corrections on explained variances. This diagram shows a single simulation rep, including repeated probe and study selections where indicated. From top to bottom, the workflow shows random selection of 500 CpG probes, random selection of 5 studies, and calculation of 3 DNAm datasets per CpG probe: (1) unadjusted DNAm; (2) DNAm after local adjustment on subsets between 1-4 study IDs among 5 selected (a.k.a. adjustment 1); (3) DNAm after uniform adjustment on all 5 selected study IDs (a.k.a. adjustment 2). Finally, ANOVAs are conducted across the 3 DNAm models, and fractions of explained variances are determined from sum of squared variances (Methods). Terminology for workflow terms is shown at top left.

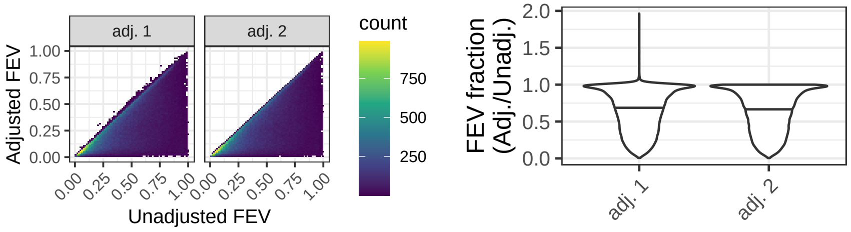

(a) (b)

Fig. S3: Fraction of explained variance (FEV) between unadjusted and adjusted DNAm across GSE bias simulations. (a) Density plots of unadjusted FEV on x axes and adjusted FEV on y-axes. Color fill shows density of simulation outcome counts (dark blue = low, green = moderate, yellow = high). (b) Violin plots of FEV fractions, or adjusted FEV over unadjusted FEV, by adjustment type on the x-axis.

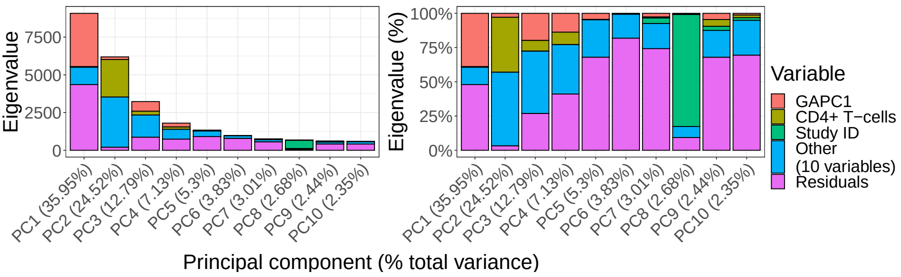

Fig. S4: Autosomal DNAm PCA results across normal blood samples. Eigenvalues explained by select variables. Stacked barplots show the eigenvalue magnitudes (left) and percentages (right) for the top ten components (x-axis). Fill colors indicate magnitudes of component sum of squared variances explained by select variables (red = genetic ancestry PC1, yellow = predicted CD4+ T-cells, green = Study ID, blue = other variable, purple = residuals). The term "other" stores the 10 remaining model variables tested. X-axis labels show the percent of total variances explained by each component in parentheses.

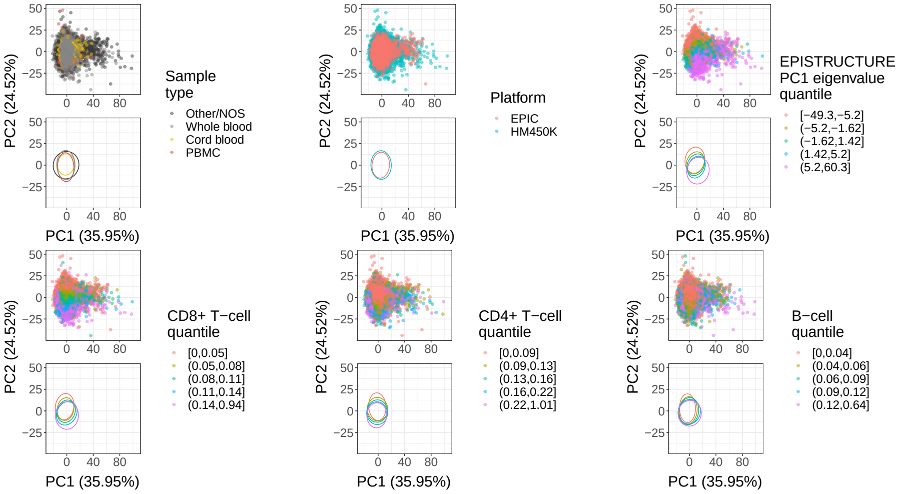

Fig. S5: Top two components from PCAs of autosomal DNAm (noob-normalized Beta-values) colored according to different variables. Each panel includes a scatter plot (top) and 95% confidence interval ellipses (bottom) where the x and y axes correspond to, respectively, the first and second components. Colors specify sample type (top left, black = other/not otherwise specified, gray = whole blood, yellow = cord blood, red = PBMC), platform (top middle, red = EPIC, blue = HM450K), the first component of genetic ancestry (top right), and predicted fractions for CD8+ T-cells (bottom left), CD4+ T-cells (bottom middle), and B-cells (bottom right). Color labels for the latter four continuous variables correspond to sample quintile bins (e.g. 5 quantile ranges: pink = 0-20, yellow = 20-40, green = 40-60, blue = 60-80, purple = 80-100).

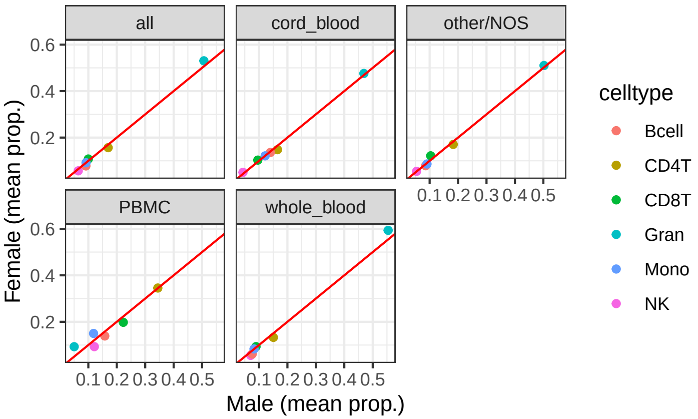

(a)

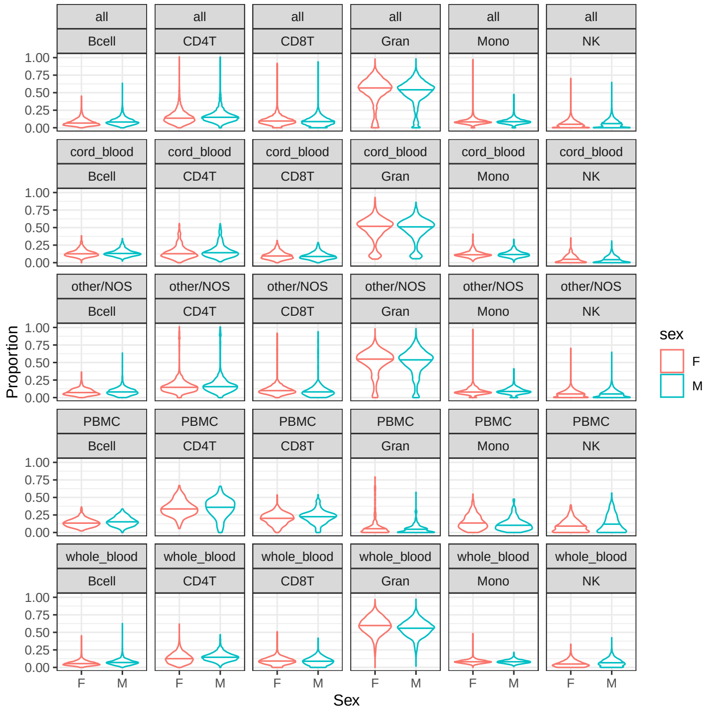

(b)

Fig. S6: Differences in blood cell mean proportions by sex. (a) Scatter plots of mean proportions of six blood cell types (points, colors) across five blood sample types (panel labels) for males (x-axes) and females (y-axes). Red diagonal lines indicate the reference (slope = 1, intercept = 0). (b) Violin plots of mean blood cell proportions in males (green distributions) and females (red distributions) across five blood cell types (rows, primary plot panel labels) and six blood cell types (columns, secondary plot panel labels). Horizontal lines represent distribution medians.

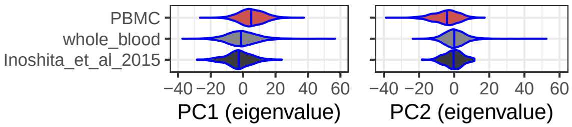

Fig. S7: Violin plots of the top two genetic ancestry components on the x axes for each of the three available datasets on the y axes (black = "Inoshita et al 2015", red = PBMC compilation, gray = whole blood compilation). Vertical blue lines represent the distribution medians.

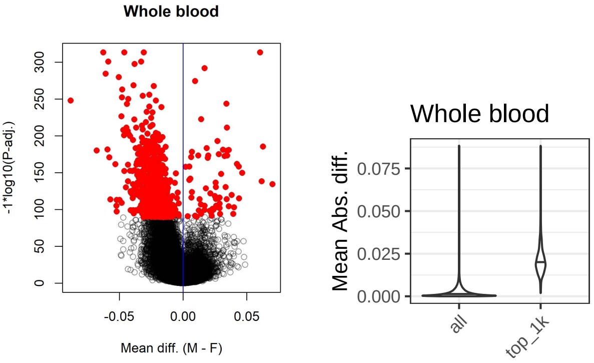

(a) (b)

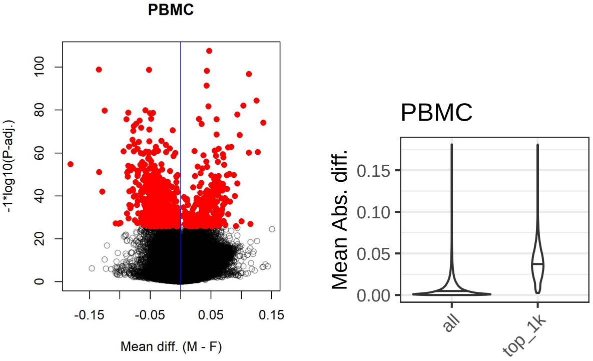

(c) (d)

Fig. S8: Differential DNAm by sex in whole blood and peripheral blood mononuclear cells (PBMC). (a) Volcano plot for whole blood showing mean Beta-value differences (males minus females, x-axis) and probe significance (-1*log10[P-adj.], Benjamini Hotchberg adjustment, y-axis). Red dots are the top 1,000 DMPs, black circles are non-DMP probes. (b) violin plots for whole blood showing absolute mean Beta-value differences (males minus females, y-axis) for all tested CpG probes (left) and only the top 1,000 DMPs (right), with medians shown in horizontal lines. (c) Volcano plot for PBMC with axes as for panel (a). Red dots are the top 1,000 DMPs, black circles are non-DMP probes. (d) violin plots with medians for PBMC, where axes are the same as for panel (b).

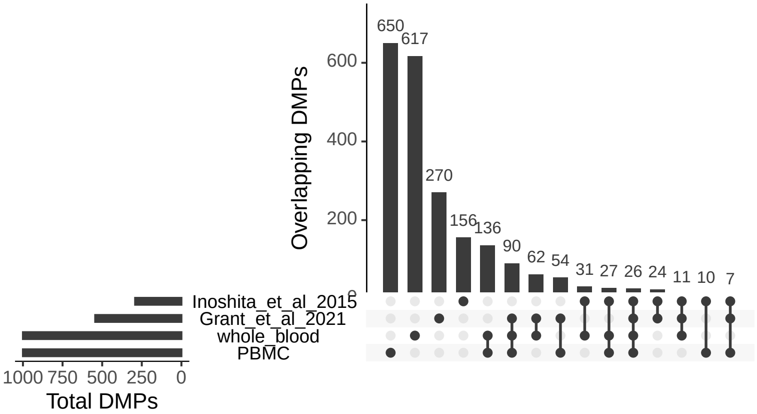

(a)

Fig. S9: Upset plot showing DMP overlaps (lower left and top right barplot magnitudes) among 4 DMP sets including compiled whole blood, compiled PBMC, "Inoshita_et_al_2015", and "Grant_et_al_2021" (lower y-axis labels). Set magnitudes are shown on top of subset magnitude barplots.
